# Forage Yield, Canopy Characteristics, and Radiation Interception of Ten Alfalfa Varieties in an Arid Environment

**DOI:** 10.3390/plants11091112

**Published:** 2022-04-20

**Authors:** Xitao Jia, Zhixin Zhang, Yanrong Wang

**Affiliations:** 1 State Key Laboratory of Grassland Agro-Ecosystems, Key Laboratory of Grassland Livestock Industry Innovation, Ministry of Agriculture and Rural Affairs, Engineering Research Center of Grassland Industry, Ministry of Education, College of Pastoral Agriculture Science and Technology, Lanzhou University, Lanzhou 730020, China; jiaxitao@gmail.com; 2 College of Grassland Agriculture, Northwest A&F University, Yangling 712100, China; zhangzhixin1986@gmail.com

**Keywords:** alfalfa, forage production, branch number, solar radiation utilization, leaf area index, light interception, arid farming systems

## Abstract

An increasing demand for new and improved livestock forage products is driving the development of forage systems in arid regions. Our study evaluated the productivity of 10 alfalfa (*Medicago sativa* L.) varieties and the relationship between forage yield and canopy structure traits, such as plant height, branch number, stem/leaf ratio, and leaf area index in the arid Hexi Corridor, north-west China. Here, plant height, primary branch number per plant, and stem/leaf ratio were positively correlated with forage yield. In terms of a two-year total yield, Gannong No. 5 produced the highest value (13,923 kg ha^−1^), followed by the WL342HQ (12,409 kg ha^−1^), Phabulous (11,928 kg ha^−1^), and Xinjiang Daye (11,416 kg ha^−1^) varieties. Therefore, these four alfalfa varieties are suitable for large-scale cultivation in the Hexi Corridor region and other arid areas where the effect of precipitation is even larger than that of temperature. These results provide valuable information for the selection and cultivation of alfalfa varieties, which could improve forage yield and the production of livestock in arid regions.

## 1. Introduction

Arid and semi-arid environments cover about 30% of the world’s total land area and 52% of that in China [1]. Additionally, the area of semi-arid land increased by 25% from 1986 to 2010 [2]. Livestock production is a major economic contributor in arid and semi-arid regions [3]. However, water is often considered to be the most important determinant that restricts forage yield in these environments [4]. Forage crops usually suffer from drought stress in these regions, even under irrigation, owing to high annual evaporation. Therefore, it is imperative to screen suitable forage crops and varieties which are more adaptable to water-limited conditions to improve livestock production sustainability in arid and semi-arid regions.

Alfalfa (*Medicago sativa* L.) is a widely planted, perennial forage legume in arid and semi-arid areas because of its high productivity, nutritional value, nitrogen fixation capacity, and adaptability to various environments [5,6]. The canopy structure has a significant influence on the forage yield of alfalfa owing to the optimization of light energy utilization and accumulation. Some traits that influence canopy development and productivity are plant height (PH), the stem to leaf ratio, branch number, leaf area index (LAI), and light interception (LI) [7,8]. Previously, a study estimated the PH of different alfalfa crop canopies, and established that PH is positively correlated with forage yield [9]. The branch number is a vital yield component of forage crops [10,11]. It exerts a strong positive impact on forage yield [12,13], and has been included in plant growth models to improve predictive ability [14]. Generally, dry matter accumulation was found to be reduced in proportion to the level of the water deficit to which the plants were exposed, because such conditions reduced shoot density and leaf area [15]. Another study shows that the number of shoots per plant varies between genotypes differing in fall dormancy [16]. Elsewhere, research has indicated that there is a positive genetic correlation between the stem/leaf ratio and forage yield [17]. A decrease in the stem/leaf ratio was found to be accompanied by reduced forage yield [18]. Thus, the stem/leaf ratio is regarded as a critical indicator of inferred forage yield. However, Annicchiarico [19] considered forage yield and leaf/stem ratio to be genetically independent, and this is consistent with genetic correlation results for alfalfa cultivars [20]. Thus, there is limited and somewhat inconsistent information on the relationship between the stem/leaf ratio and forage yield of alfalfa. The LAI is a useful indicator that affects forage yield; it has been used indirectly as an input variable for primary production models, crop growth, and yield forecasting models [21,22,23,24,25]. The LI reflects the photosynthetically active radiation intercepted and accumulated during the crop growth cycle which is converted into biomass [26,27]. Therefore, LAI and LI should be considered when investigating forage productivity.

Recently, several studies have assessed alfalfa varieties for their morphological characteristics and forage yield under drought conditions. For instance, among Mediterranean cultivars, Ameristand801S and ABT805, for example, were found to be more drought tolerant than Magali, Melissa, and Coussouls, owing to higher persistence values and autumn dormancy [28]. Other cultivars such as Anchor and WL 318 are considered drought-resistant in the U.S. owing to their greater root length and water retention [29]. In France, the TC cultivar has been planted in arid regions owing to its high water use efficiency, leaf area ratio, specific leaf area, and dry matter content [30]. In China, cultivars such as Xinmu No. 1, Longdong, and Aohan showed a high degree of tolerance to drought stress [31,32,33]. Nevertheless, there is a need to find other varieties that have better performance in order to sustain optimal productivity of foraging agricultural systems in these regions.

The Hexi Corridor is an arid region located in the Gansu Province (north-western China) with abundant light and heat resources for crop production [34,35]. However, the forage growing season is generally short, resulting in a severe seasonal imbalance in forage yield, and challenging livestock production. This is further aggravated by the degradation of natural grassland caused by high livestock density grazing and the lack of research-based planning during grazing. These factors result in low and unstable forage production that cannot meet the yield and nutritional demands of livestock. Therefore, it is necessary to select forage species and varieties with high yield and quality. In 2013, the local government identified alfalfa as a suitable forage option; it was subsequently grown on about 650,000 ha in Gansu province [36]. Currently, the Xinjiang Daye variety is widely grown in this region [37]. However, information on other varieties suitable for planting in the region is insufficient. Thus, selecting more suitable alfalfa varieties is essential for the development of forage and improvement of livestock production.

To understand the conditions of alfalfa growth, we hypothesized that canopy structure traits were critical indicators for forage yield. This study explored the morphological traits and productivity of 10 alfalfa varieties in the Hexi Corridor. The study objectives were to: (1) evaluate the effects of canopy structure traits on forage yield; and (2) identify alfalfa varieties which are most suitable for arid regions. The results will guide alfalfa production and variety development in dry areas.

## 2. Results

### 2.1. Climatic Conditions

**Figure 1:**
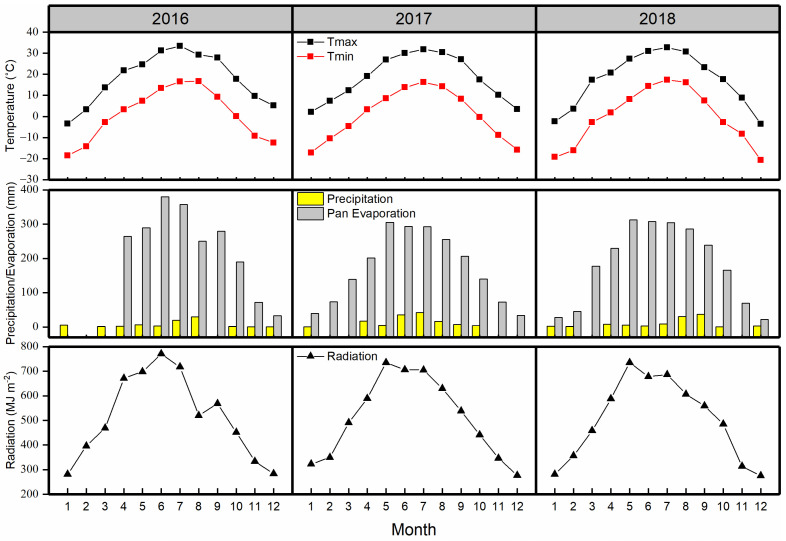
Monthly maximum (Tmax), and minimum (Tmin) average air temperature (°C), precipitation (mm), pan evaporation (mm), and cumulative radiation (MJ m^−2^) at the experimental station in 2016–2018.

Precipitation varied in the three study years. In 2017, the annual precipitation was 127 mm, i.e., higher than the long-term average (117 mm; 2005–2020), while in 2016 and 2018 (69 and 99 mm, respectively) it was lower. From 2016 to 2018, precipitation within the growing season (April to October) was 61, 126, and 93 mm, respectively. However, 75–79% of precipitation occurred during four months (June to September) within the experimental years (Figure 1). In terms of pan evaporation, the values were 2116 mm in 2016, 2055 mm in 2017, and 2187 mm in 2018. Overall, the annual pan evaporation was 20 times greater than the annual precipitation between 2016 and 2018.

In 2016, 2017, and 2018, the average air temperatures were 18.4 °C, 18.0 °C, and 17.8 °C, respectively. The maximum average air temperature occurred in July 2016 (33.4 °C), 2017 (31.8 °C), and 2018 (32.8 °C), and the warmest months were June, July, and August. During the growing seasons, the accumulated radiation was 6160 MJ m^−2^, 6131 MJ m^−2^, and 6021 MJ m^−2^ in 2016, 2017, and 2018, respectively. As demonstrated in Figure 1, the maximum monthly cumulative radiation value was recorded in June for 2016 and in May for 2017 and 2018.

### 2.2. Forage Yield

**Figure 2:**
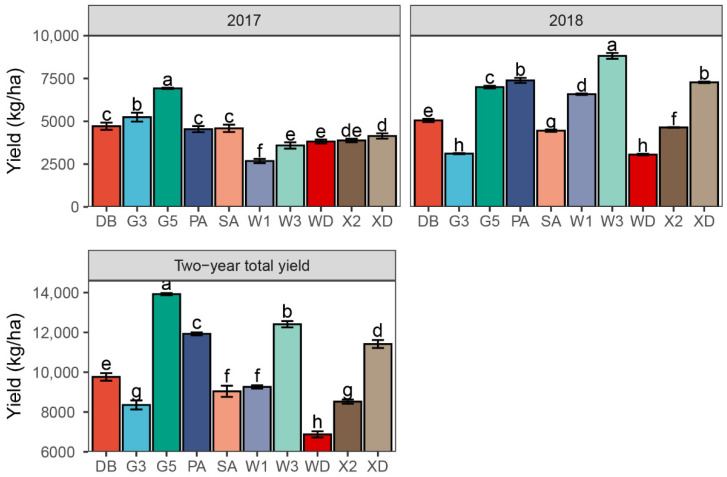
The annual yield of 10 alfalfa varieties in 2017 and 2018, and the two-year total yield. Different letters indicate means that are significantly different at *p* < 0.05. Error bars indicate the standard deviation of the means. DB: Derby, G3: Gannong No. 3, G5: Gannong No. 5, PA: Phabulous, SA: Sanditi, W1: WL168HQ, W3: WL343HQ, WD: Wudi, X2: Xinmu No. 2, XD: Xinjiang Daye.

The interaction between year and variety significantly influenced annual yield and the yield of each cutting (*p* < 0.001) (Table 1). From 2017 to 2018, a significant increase in performance was observed in WL168HQ (*p* < 0.001), WL343HQ (*p* < 0.001), Xinjiang Daye (*p* < 0.001), Phabulous (*p* < 0.001), and Xinmu No. 2 (*p* < 0.001) (Figure 2, Table S1); the rates of increase were 146%, 146%, 76%, 63%, and 20%, respectively (Figure 2). In contrast, we detected an annual yield reduction rate of 41% and 20% in the Gannong No. 3 (*p* = 0.004) and Wudi (*p* < 0.001) varieties, respectively. Lastly, there was no significant difference in the annual yields of the Derby, Gannong No. 5, and Sanditi varieties (*p* = 0.063, 0.197, and 0.353, respectively) between 2017 and 2018.

In 2017, the annual yield of Gannong No. 5 (6924 kg ha^−1^) was significantly higher than those of all the other alfalfa varieties, followed by Gannong No. 3 (5242 kg ha^−1^) (Figure 2, *p* < 0.001). In addition, we observed the lowest annual yield in WL168HQ (2680 kg ha^−1^) (*p* < 0.001). In 2018, the annual yield varied from 3057 kg ha^−1^ in Wudi to 8820 kg ha^−1^ in WL343HQ (*p* < 0.001). In terms of the two-year total yield, Gannong No. 5 (13,923 kg ha^−1^) had the greatest significant value among the 10 varieties, followed by WL343HQ (12,409 kg ha^−1^), Phabulous (11,928 kg ha^−1^), Xinjiang Daye (11,416 kg ha^−1^), and Derby (9762 kg ha^−1^) (*p* < 0.001), whereas the lowest value was observed with the Wudi variety (6876 kg ha^−1^) (*p* < 0.001).

**Figure 3:**
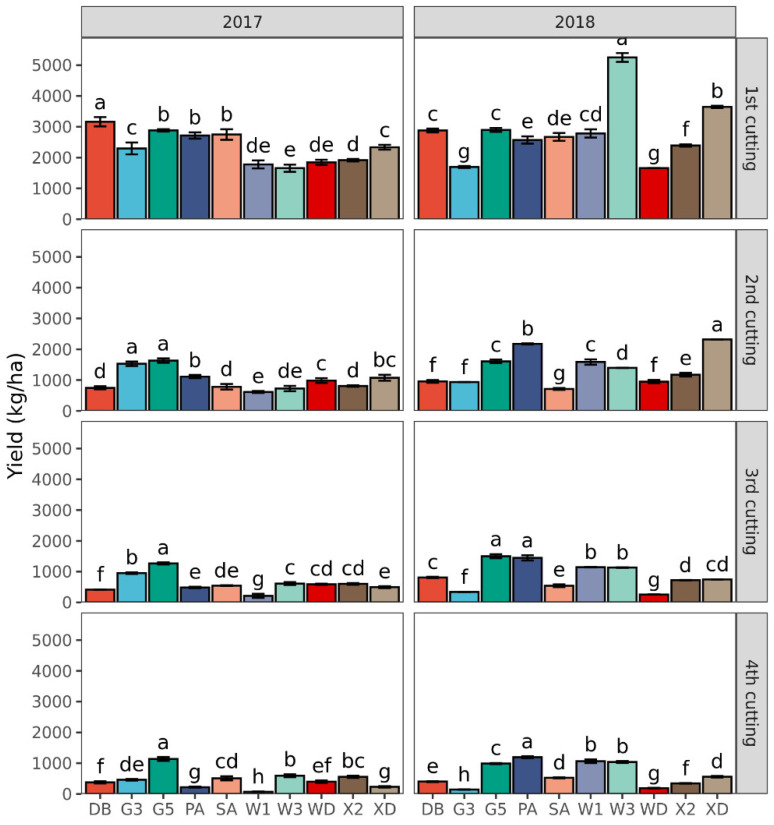
Forage yield for each cutting (1st to 4th) of 10 alfalfa varieties in 2017 and 2018. Different letters indicate means that are significantly different at *p* < 0.05. Error bars indicate the standard deviation of the means. DB: Derby, G3: Gannong No. 3, G5: Gannong No. 5, PA: Phabulous, SA: Sanditi, W1: WL168HQ, W3: WL343HQ, WD: Wudi, X2: Xinmu No. 2, XD: Xinjiang Daye.

The forage yield in each cutting decreased with harvest time (Figure 3). In 2017, the proportion of first cutting yield to annual yield of 10 varieties ranged from 42 to 67%, whereas in 2018, it ranged from 35 to 60%. In addition, the first cutting yield of Derby (3163 kg ha^−1^) was significantly the greatest among the 10 varieties, followed by Gannong No. 5 (2883 kg ha^−1^), Sanditi (2748 kg ha^−1^), and Phabulous (2716 kg ha^−1^) (*p* < 0.001) in 2017. The second cutting yield varied from 615 kg ha^−1^ in WL168HQ to 1633 kg ha^−1^ in Gannong No. 5 (*p* < 0.001) in 2017. On average, the third and fourth cutting yields were equivalent to 27% (619 kg ha^−1^) and 20% (457 kg ha^−1^) of the first cutting yield (2334 kg ha^−1^) of the 10 alfalfa varieties in 2017. In 2018, the top two yields of the first cutting were WL343HQ (5251 kg ha^−1^) and Xinjiang Daye (3648 kg ha^−1^) varieties, whereas the lowest value was observed with Gannong No. 3 (1694 kg ha^−1^) and Wudi (1657 kg ha^−1^) varieties (*p* < 0.001). Here, we observed that the second cutting yield of Xinjiang Daye (2318 kg ha^−1^) was higher than those of the other nine varieties, while the lowest values occurred with Sanditi (713 kg ha^−1^) (*p* < 0.001).

### 2.3. Plant Height

Plant height at spring (PHS) and at flowering (PHF) were measured in this study. These parameters were found to be significantly affected by the varieties and the interaction between years and varieties (*p* < 0.001) (Table 1). In 2017, the PHSs of Gannong No. 5 (26.6 cm) and Derby (25.3 cm) were significantly greater than those of other alfalfa varieties, whereas the lowest values were observed in WL168HQ (17.6 cm) and Wudi (17.8 cm). In 2018, the greatest and lowest PHSs occurred in Gannong No. 5 (29.2 cm) and Sanditi (16.8 cm), respectively. Overall, Phabulous and WL343HQ presented a significant increase in PHS from 2017 to 2018 (Table S1, *p* = 0.047, and =0.012, respectively). In contrast, Sanditi showed a significant decrease (*p* = 0.02).

**Figure 4:**
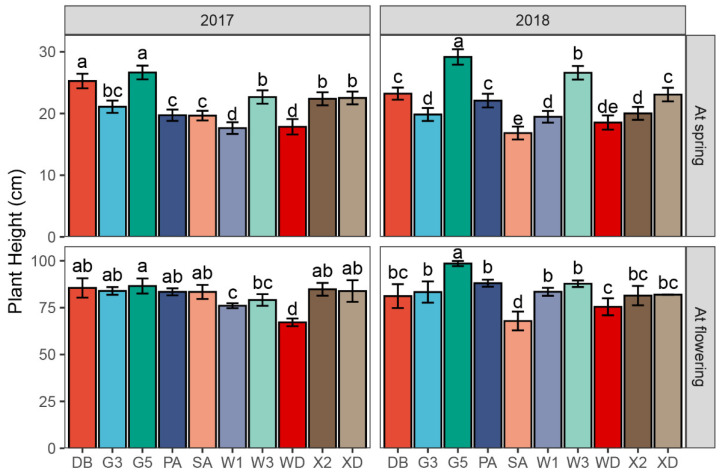
The plant heights of 10 alfalfa varieties at spring and at flowering of first cutting in 2017 and 2018. Different letters indicate significantly different means at *p* < 0.05. Error bars indicate the standard deviation of the means. DB: Derby, G3: Gannong No. 3, G5: Gannong No. 5, PA: Phabulous, SA: Sanditi, W1: WL168HQ, W3: WL343HQ, WD: Wudi, X2: Xinmu No. 2, XD: Xinjiang Daye.

In terms of PHF, the values of Gannong No. 5, Phabulous, WL168HQ, WL343HQ, and Wudi were significantly higher in 2018 than in 2017 (Table S1, *p* = 0.008, 0.039, 0.007, 0.014, and 0.045, respectively). However, the PHF of Sanditi was significantly lower (*p* = 0.013). There were no significant differences for Derby, Gannong No. 3, Xinjiang Daye, and Xinmu No. 2 between years (Figure 4, Table S1, *p* = 0.408, 0.871, 0.621, and 0.399, respectively). In 2017, the PHF of Wudi (67.2 cm) was considerably lower than those of the other nine alfalfa varieties (*p* < 0.001). In 2018, the lowest and greatest PHFs occurred in Sanditi (67.9 cm) and Gannong No. 5 (98.5 cm), respectively (*p* < 0.001). Additionally, the PHF of Wudi (75.5 cm) was lower than those of Gannong No. 3 (83.3 cm), Phabulous (88.1 cm), WL168HQ (83.5 cm), and WL343HQ (87.8 cm) (*p* < 0.001).

### 2.4. Branch Number

**Figure 5:**
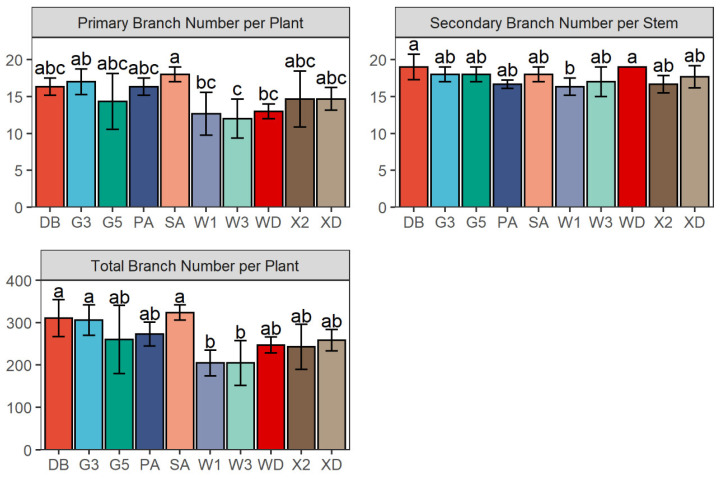
The Primary Branch number per plant (PB), Secondary Branch number per stem (SB), and Total Branch number per plant (TB) of 10 alfalfa varieties at the flowering stage before the first cutting in 2017. Different letters indicate means that are significantly different at *p* < 0.05. Error bars indicate the standard deviation of the means. DB: Derby, G3: Gannong No. 3, G5: Gannong No. 5, PA: Phabulous, SA: Sanditi, W1: WL168HQ, W3: WL343HQ, WD: Wudi, X2: Xinmu No. 2, XD: Xinjiang Daye.

As illustrated in Figure 5, the number of branches varied among the 10 varieties in 2017. The primary branch number per plant (PB) of Sanditi (18.0) was higher than those of WL168HQ (12.7), Wudi (13.0), and WL343HQ (12.0) (*p* = 0.079). The secondary branch number per stem (SB) of Derby (19.0) and Wudi (19.0) were significantly greater than that of WL168HQ (16.3) (*p* = 0.131). Lastly, the total branch number per plant (TB) of Derby (311), Gannong No. 3 (306), and Sanditi (324) were significantly higher than those of WL168HQ (205) and WL343HQ (205) (*p* = 0.026).

### 2.5. Leaf Area Index and Light Interception

**Figure 6:**
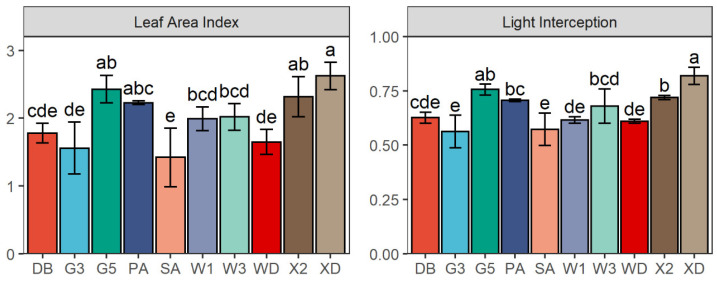
Leaf area index (LAI) and light interception (LI) of 10 alfalfa varieties at the flowering (first cutting) in 2017. Different letters indicate means that are significantly different at *p* < 0.05. Error bars indicate the standard deviation of the means. DB: Derby, G3: Gannong No. 3, G5: Gannong No. 5, PA: Phabulous, SA: Sanditi, W1: WL168HQ, W3: WL343HQ, WD: Wudi, X2: Xinmu No. 2, XD: Xinjiang Daye.

The LAI varied significantly among the 10 alfalfa varieties (*p* < 0.001) (Figure 6). The LAI of Xinjiang Daye, Gannong No. 5, and Xinmu No. 2 (2.62, 2.43, and 2.32, respectively) were significantly higher than those of Derby (1.78), Gannong No. 3 (1.56), Sanditi (1.42), and Wudi (1.65) (*p* < 0.001). Alternatively, the LI of the Xinjiang Daye varieties was significantly greater than that of Xinmu No. 2 (*p* < 0.001). However, the LI of Gannong No. 3 and Sanditi (0.56 and 0.57, respectively) were significantly lower than those of Gannong No. 5 (0.76), Phabulous (0.71), WL343HQ (0.68), Xinmu No. 2 (0.72), and Xinjiang Daye (0.82) (*p* < 0.001).

### 2.6. Stem to Leaf Ratio

**Figure 7:**
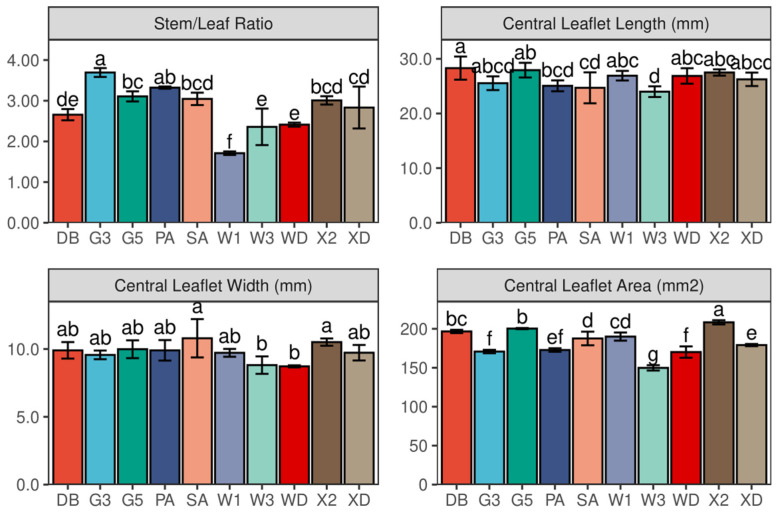
Stem/Leaf Ratio and central Leaflet Length, Width, and Area at flowering (first cutting) in 2017. Different letters indicate means that are significantly different at *p* < 0.05. Error bars indicate the standard deviation of the means. DB: Derby, G3: Gannong No. 3, G5: Gannong No. 5, PA: Phabulous, SA: Sanditi, W1: WL168HQ, W3: WL343HQ, WD: Wudi, X2: Xinmu No. 2, XD: Xinjiang Daye.

As shown in Figure 7, the stem to leaf ratio varied among the 10 varieties in 2017. The stem/leaf ratio of Gannong No. 3 (3.69) was significantly greater than those of the other eight alfalfa varieties, except Phabulous (*p* < 0.001). The lowest ratio was observed in WL168HQ (1.71) (*p* < 0.001).

### 2.7. The Central Leaflet Length, Width, and Area

The leaf size attributes (length, width, and area) differed significantly among the 10 varieties (*p* = 0.035, 0.013, and <0.001, respectively) (Figure 7). The central leaflet length varied from 24.0 mm (WL343HQ) to 28.3 mm (Derby) (*p* = 0.035). Its width varied from 8.7 mm (Wudi) to 10.8 mm (Sanditi) (*p* = 0.013). The central leaflet area of Xinmu No. 2 had the greatest value (208 mm^2^) among 10 alfalfa varieties (*p* < 0.001). Moreover, the area of central leaflet of WL343HQ (150 mm^2^) was significantly lower than those of other alfalfa varieties (*p* < 0.001).

### 2.8. Correlation Analysis for First Cutting Yield

**Figure 8:**
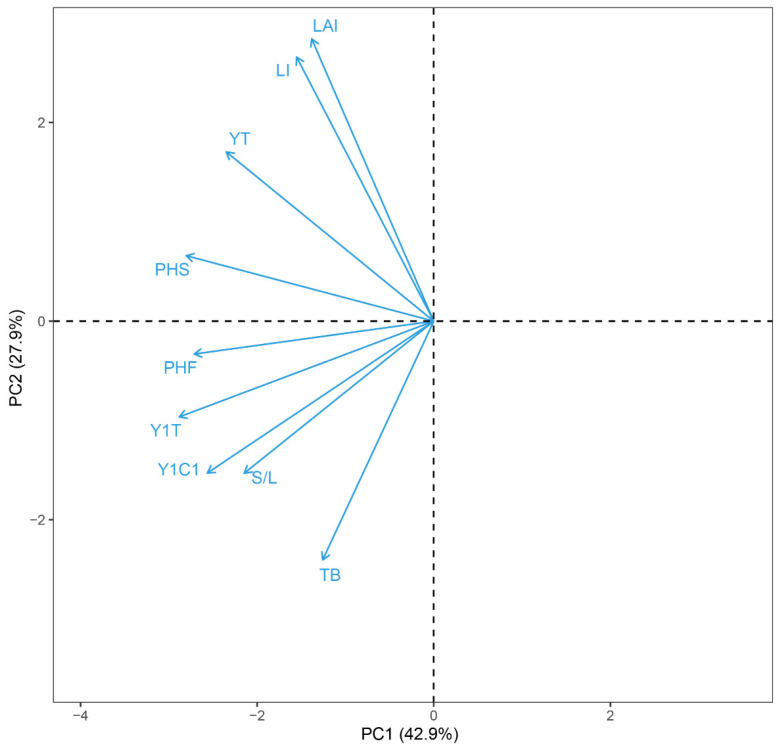
Principal component analysis of alfalfa growth traits and forage yield. PHS: plant height at spring, PHF: plant height at flowering of first cutting, TB: total branch number per plant, LAI: leaf area index, LI: light interception, S/L: stem to leaf ratio, Y1C1: first cutting yield in 2017, Y1T: total yield from four cuttings in 2017, YT: two-year total yield.

Considering that forage yield was influenced by the morphological traits of the plants, we analyzed correlations between forage yield and the above-mentioned traits using data (sample size of 30 for each trait) in the same growing year (2017), which are summarized in the correlation coefficient matrix (Table 2) and PCA biplot based on the correlation matrix (Figure 8). Before starting PCA, the applicability of PCA was tested using the Kaiser–Meyer–Olkin (KMO) and Barlett tests. The calculated results were KMO = 0.66 (>0.5) and Barlett test value = 0 (<0.05), indicating that the dataset was appropriate for a factor analysis.

The first two principal components explained 70.8% of the first yield variation (Figure 8). Thus, we focused on these two components to represent the PCA ordination biplot loadings. The angles between the vectors represent the correlations, where small angles (close to 0°) indicate high positive correlations; wide angles (close to 180°) indicate high negative correlations; and angles close to 90° indicate a lack of correlation [38]. As illustrated in Figure 8 and Table 2, PCA and correlation analysis showed that the yield of first cutting had a significant positive association with PHS, PHF, and TB (*p* = 0.006, 0.002, and <0.001, respectively) in 2017. The total yield of 2017 was strongly related to PHS, PHF, and stem/leaf ratio (*p* < 0.001, =0.005, and <0.001, respectively). The two-year total yield was significant positive affected by PHF, LAI, and LI (*p* < 0.001, =0.002, and <0.001, respectively). Moreover, there was a highly significant positive relationship between LAI and LI (r = 0.8745, *p* < 0.001) (Table 2). The stem/leaf ratio showed a significant positive association with PHF and PB (*p* < 0.001 and =0.027, respectively).

## 3. Discussion

Stem traits, including plant height (PH), branch number, and stem to leaf ratio, are essential indicators related to the growth and development of forage, and are associated with the distribution of solar radiation in the canopy of forage crops [39]. In this study, PH (including PHS and PHF) was shown to significantly affect forage yield, which is consistent with previous studies [9,20,40]. In addition, PH demonstrated a positive correlation to the central leaflet area and stem to leaf ratio, but no effects on LAI and LI.

Branch number is an important morphological characteristic that could enhance the forage yield in alfalfa grown in arid environments [16]. In this study, the results indicated that PB was significantly corelated with forage yield, whereas SB had no effect on yield. Additionally, SB expressed less internal variation among the 10 alfalfa varieties. These results demonstrated that PB plays a greater role in plant architecture construction, determining forage yield. These findings are consistent with those of previous studies [41]. Therefore, we suggest that PB should be viewed as a crucial indicator of high productivity varieties for plant breeding research in the future.

The PCA and correlation analysis results revealed that a higher stem/leaf ratio indicates a better yield performance. This could be attributed to a higher stem/leaf ratio accompanied by higher PH and PB. These results are consistent with those reported by other researchers [17,18,42].

The leaf is considered to be an important functional unit of a plant, contributing to its yield formation. Traditionally, LAI and LI demonstrated a positive correlation with forage yield [25]. In this study, LAI and LI had a significant positive correlation with two-year total yield. However, they did not affect the yield of first cutting and total yield from four cuttings in 2017. The first reason could be that the effects of leaflet density and number to the LAI and LI were more significant than the central leaflet size in an arid environment [43]. Thus, in arid environments, the positive yield effect of canopy structure traits should not be attributed simply to the leaf traits, such as LAI and LI; rather, it could also be due to its effects of stem traits, like PH and branch number, via an increase in the stem dry weight ratio, as proposed by Hakl (2021) [7] and Ta (2020) [8]. Secondly, forage yield is related to light interception that accumulates throughout the entire growth cycle. Therefore, only one LAI and LI measurement before harvest does not truly reflect the efficiency of light energy utilization. It is suggested that researchers consider the significance of dynamic and continuous measurements of LAI and LI in future experiments.

Choosing an alfalfa variety is arguably one of the most important decisions to make in an alfalfa operation. Alfalfa yield reveals the suitability of a given variety to arid regions. On average, the forage annual yield in the second harvest year increased by 30% (1326 kg ha^−1^) compared with that of the first harvest year. Similarly, a study showed that the forage yield of alfalfa reached peak performance in the second and third harvest years [44]. Therefore, the advantage of accumulation of perennial alfalfa biomass appeared in the second harvest year. From our perspective, we should not only consider the yield of first harvest year, but also pay close attention to long-term productivity.

As one of the most widely planted varieties in the Hexi Corridor, Xinjiang Daye showed good forage yield in 2017 and 2018. In addition, each yield of its four cuttings significantly increased between the two growing years. In addition, it showed outstanding performance regarding the accumulation of light energy owing to its high leaf area index and excellent light interception.

Three varieties, i.e., Gannong No. 5, WL343HQ, and Phabulous, performed better in terms of their two-year total yield than Xinjiang Daye. Gannong No. 5 gave the highest value, i.e., 22% (2507 kg ha^−1^) higher than that of Xinjiang Daye. This could be due to its excellent PH, LAI, LI, central leaflet area, and stem to leaf ratio. WL343HQ presented the most significant increase in forage yield from 2017 to 2018 among the 10 alfalfa varieties, and reached the highest yield in 2018. The two-year total yield of WL343HQ was more than 9% (993 kg ha^−1^) higher than that of Xinjiang Daye. WL343HQ had a low PH, low PB, low stem/leaf ratio and low central leaflet area, implying that this cultivar has substantial nutritional potential. A higher leaf/stem ratio has been largely used as a positive indicator of alfalfa forage quality in previous studies [19], owing to its strict positive correlation with digestibility and intake of forage [18] that arises from the higher digestibility of leaves relative to stems [45,46]. The two-year yield of Phabulous was 4% (512 kg ha^−1^) more than that of Xinjiang Daye. Phabulous possessed a high potential for forage yield because of its high stem/leaf ratio and PH features. However, the two-year total yield of Derby was slightly below 14% (1653 kg ha^−1^) compared with that of Xinjiang Daye. Considering its high branch number, PH, and the central leaflet area, Derby could be used as a supplementary variety to reduce forage shortages for animals.

In 2017 and 2018, Wudi displayed poor performances in its annual yield. Gannong No. 3 showed poor productivity, significantly reducing each cutting yield from 2017 to 2018. Although the yield of Xinmu No. 2 and WL168HQ increased from 2017 to 2018, they presented poor performances in their two-year total yields. Moreover, Wudi, Sanditi, Gannong No. 3, Xinmu No. 2, and WL168HQ showed poor over-wintering performances (data not shown) in this study. When winter hardiness is poor for alfalfa, the productivity is reduced in an arid environment [47]. Generally, the yield should be the greatest determining factor for variety selection. In this study, Wudi, Gannong No. 3, Xinmu No. 2, Sanditi, and WL168HQ were found to be the least suitable varieties and are not recommended for planting in the Hexi Corridor region.

The study evaluated the correlation of forage yield with canopy structure traits and analyzed the productivity of 10 alfalfa varieties in an arid environment. Our results suggest that we should focus more intensively on stem traits, such as PH, PB, and stem/leaf ratio, which contribute significantly to canopy structure, and consequently, produce more forage yield in an arid environment. Moreover, Gannong No. 5, WL343HQ, Phabulous, and Xinjiang Daye achieved the highest forage yields. However, more field experiments with multiple locations are necessary to test these varieties over a broader area. In addition, a closer investigation of forage quality and related indicators of the selected varieties is necessary in follow-up research. These results provide more insights into alfalfa characteristics and could be used to improve forage yield and livestock production sustainability in the world’s ever-expanding arid regions.

## 4. Materials and Methods

### 4.1. Site Description

The field experiment was conducted from 2016 to 2018 at the Linze experimental station of Lanzhou University in the Hexi Corridor, Gansu Province, China (100°02′ E, 39°15′ N, elevation 1390 m a.s.l.), a typical temperate desert climate area. We collected the climate datasets from 2005 to 2020. The annual mean air temperature was 7.6 °C, absolute maximum and minimum were 39.1 °C and −27 °C, and average annual precipitation was 117 mm. Moreover, the mean annual pan evaporation was 2390 mm, i.e., 20 times greater than the annual average precipitation. The frost-free season was 165 days, on average. The annual accumulated temperature of ≥10 °C was 3088 °C, on average [48].

The experimental site has a meadow soil mapped as an Entisol according to the U.S. soil classification system [49], a pH of 7.6, 0.806g kg^−1^ total nitrogen, and 0.703 g kg^−1^ total phosphorus at sowing in 2016. Previously, the experimental site had been utilized to grow yellow sweet clover [*Melilotus officinalis* (L.) Pall.] and maize (*Zea mays* L.).

### 4.2. Experimental Design and Agronomic Management

As outlined in Table 3, a total of 10 alfalfa cultivars were used in this experiment (Table 3). Alfalfa seeds were sown in nursery substrate (containing more than 40% organic matter and 15% humic acid) and acclimatized in a greenhouse for 45 days. Each plant was then transplanted into the experimental site on 20 June 2016. All experimental plots were arranged in a randomized complete block design with three replicates. The plot size was 3.6 m × 2.4 m, and the seedlings were planted at a spacing of 60 cm within and between rows.

In 2016, the experimental field received rotary tillage to a depth of 0.2 m before sowing. In addition, no fertilizer was applied at sowing or during the growing seasons of this experiment. The field received 150 mm of irrigation on 24 April, 25 June, 25 August, and 25 October during each growing season based on conventional local practices. However, irrigation could not compensate for the water loss caused by high pan evaporation (over 2390 mm per year). Weeds were controlled manually as necessary.

### 4.3. Measurements

#### 4.3.1. Meteorological Data

Daily precipitation, incoming solar radiation, and maximum and minimum air temperature data were obtained from the Linze Farming Ecosystem experimental weather station of the Chinese Academy of Sciences [50].

#### 4.3.2. Plant Height

Plant Height was measured one month after the spring emergence and at flowering (first cutting). Three plants were randomly selected from each plot, and their height was measured as the distance from ground to the top end using a ruler (accuracy 1 mm).

#### 4.3.3. Branch Number

During the initial growth phase, a primary axis develops from the seed, and secondary shoots subsequently develop from the axillary buds of cotyledons and primary axes leaves [51]. The primary branch number per plant (PB) and secondary branch number per stem (SB) were counted on five randomly selected plants from each plot at the flowering stage before the first cutting in 2017. Lastly, the total branch number per plant (TB) was calculated as the product of PB and SB.

#### 4.3.4. Leaf Area Index and Light Interception

The leaf area index (LAI) and light interception (LI) were measured at the flowering stage before the first cutting in 2017 during clear sunshine from 11:00 to 13:00 using a Ceptometer (AccuPAR LP-80, Decagon Devices Inc., Pullman, WA, USA). One above and four below the canopy (close to the ground) randomly chosen readings on points were collected from each plot. Notably, the average below canopy reading was used. The *LI* was calculated [52] as:

(1) $$LI=1-\frac{PAR_{bl}}{PAR_{ab}}\;,$$

where *PAR_bl_* and *PAR_ab_* are photosynthetically active radiation below and above the canopy, respectively.

#### 4.3.5. Forage Yield and Stem to Leaf Ratio

Samples were harvested manually using scythes on the plant base and measured four times annually in 2017 and 2018 at an interval of 4–5 weeks, starting at the initial flowering stage, specifically, 2 June, 8 July, 9 August, and 14 September in 2017, and 12 June, 14 July, 17 August, and 18 September in 2018. In the first cutting of 2017, the stem/leaf ratio was recorded on a single plant basis (after separation and oven drying of the stems and leaves). Subsequently, 10 plants were randomly chosen from each plot, cut at ground level and immediately weighed, before being oven-dried at 64 °C for 48 h to a constant weight in order to determine the dry weight.

#### 4.3.6. The Central Leaflet Length, Width, and Area

The central leaflet was a mature leaf taken from the main stem. To measure the length, width, and area, 20 randomly selected central leaflets from each plot (each species a total of 60 leaflets) were scanned, and the image was analyzed with an Epson GT-15000 and imaging software (WinSEEDLETM, Quebec City, QC, Canada).

### 4.4. Statistical Analysis

All the statistical analyses described below were performed using the R software (version 4.0.3). Plots were produced using the ggplot2 [53] and Factoextra [54] packages. A one-way analysis of variance (ANOVA) was conducted to assess differences of all measured traits among the 10 alfalfa varieties during the experimental years. The main effects and interactions of year and cultivar were obtained using two-way ANOVA. All tests were considered significantly different at *p* < 0.05, and the means were compared using Duncan’s multiple comparison test. In addition, an independent samples t-test was conducted to assess the differences of traits between 2017 and 2018. Finally, we evaluated the relationships between canopy traits and forage yield using principal component analysis (PCA) based on a correlation matrix and Pearson’s correlation analysis. Before starting PCA, we checked the sampling adequacy to detect whether or not the data would factor well. Sampling adequacy was measured by the Kaiser–Meyer–Olkin criterion (KMO). KMO varied from 0 to 1 and the overall KMO had to be not less than 0.5 to proceed to the principal component analysis. The Bartlett Test is another criterion in this sampling procedure which is likely also significant [55].

## Figures and Tables

**Table 1:** The two-way ANOVA *p*-values for main effects and interactions of year and cultivar on alfalfa yield and plant height.

	DF	PHS	PHF	1st Yield	2nd Yield	3rd Yield	4th Yield	Annual Yield
Year	1	0.224	0.125	<0.001	<0.001	<0.001	<0.001	<0.001
Variety	9	<0.001	<0.001	<0.001	<0.001	<0.001	<0.001	<0.001
Year × variety	9	<0.001	<0.001	<0.001	<0.001	<0.001	<0.001	<0.001

Note: DF: degrees of freedom, PHS: plant height at spring, PHF: plant height at flowering of first cutting, 1st Yield: first cutting yield, 2nd Yield: second cutting yield, 3rd Yield: third cutting yield, 4th Yield: fourth cutting yield, Annual Yield: total yield from four cuttings.

**Table 2:** Correlation coefficient matrix among morphological traits of 10 alfalfa varieties.

	PHS	PHF	PB	SB	LAI	LI	S/L	Y1C1	Y1T	YT
PHS	1									
PH	0.577 ***	1								
PB	0.018	0.300	1							
SB	0.125	−0.011	0.100	1						
LAI	0.386	0.242	−0.213	−0.343	1					
LI	0.425	0.206	−0.119	−0.157	0.875 ***	1				
S/L	0.295	0.574 ***	0.403 *	0.129	−0.023	−0.039	1			
Y1C1	0.493 **	0.548 **	0.55 **	0.273	−0.07	0.018	0.462	1		
Y1T	0.648 ***	0.495 **	0.332	0.251	0.067	0.137	0.652 ***	0.702 ***	1	
YT	0.587 ***	0.45	−0.092	−0.192	0.552 **	0.576 ***	0.111	0.32	0.455	1

Note: *, ** and *** denote significance difference at *p* < 0.05, 0.01 and 0.001, respectively. PHS: plant height at spring, PHF: plant height at flowering of first cutting, PB: primary branch number per plant, SB: secondary branch number per stem, LAI: leaf area index, LI: light interception, S/L: stem leaf ratio, Y1C1: first cutting yield in 2017, Y1T: total yield from four cuttings in 2017, YT: two-year total yield.

**Table 3:** Details of 10 alfalfa varieties used in this study.

Name	Type	Country of Origin	Institution-Provided Seed
Derby	Cultivar	United Kingdom	Lanzhou University
Gannong No. 3	Cultivar	China	Gansu Agriculture University
Gannong No. 5	Cultivar	China	Gansu Agriculture University
Phabulous	Cultivar	United States	USDA-NPGS
Sanditi	Cultivar	France	Lanzhou University
WL168HQ	Cultivar	United States	USDA-NPGS
WL343HQ	Cultivar	United States	USDA-NPGS
Wudi	Land race	China	Lanzhou University
Xinjiang Daye	Land race	China	Lanzhou University
Xinmu No. 2	Cultivar	China	Lanzhou University

Note: USDA-NPGS: United States Department of Agriculture, National Plant Germplasm System.

## Data Availability

All the data supporting this study are included in the article.

## Supplementary Materials

The following supporting information can be downloaded at: https://www.mdpi.com/article/10.3390/plants11091112/s1, Table S1: *p*-values for independent sample *t*-tests of 10 alfalfa varieties between 2017 and 2018.
