# Supplementary material for: Forage Yield, Canopy Characteristics, and Radiation Interception of Ten Alfalfa Varieties in an Arid Environment
Source: Plants (Basel). 2022 Apr 20;11(9):1112. doi: 10.3390/plants11091112 (PMC9101258; doi:10.3390/plants11091112)
Supplement: Supplementary file 1 [file plants-11-01112-s001.zip › plants-1644458-supplementary.pdf]

Table S1. *P*-values for independent sample *t*-tests of 10 alfalfa varieties between 2017 and 2018.

	PHS	PHF	1stYield	2ndYield	3rdYield	4thYield	Annual Yield
Derby	0.082	0.408	0.040	0.005	< 0.001	0.358	0.063
Gannong No. 3	0.209	0.871	0.006	< 0.001	< 0.001	< 0.001	0.004
Gannong No. 5	0.060	0.008	0.759	0.686	0.005	0.016	0.197
Phabulous	0.047	0.039	0.186	< 0.001	< 0.001	< 0.001	< 0.001
Sanditi	0.020	0.013	0.563	0.268	0.895	0.699	0.353
WL168HQ	0.770	0.007	< 0.001	< 0.001	0.002	< 0.001	< 0.001
WL343HQ	0.012	0.014	< 0.001	0.006	< 0.001	< 0.001	< 0.001
Wudi	0.521	0.045	0.062	0.566	0.001	< 0.001	< 0.001
Xinjiang Daye	0.565	0.621	< 0.001	< 0.001	0.005	< 0.001	< 0.001
Xinmu No. 2	0.051	0.399	< 0.001	< 0.001	0.001	< 0.001	< 0.001

Note: PHS: plant height at spring, PHF: plant height at flowering of first cutting, 1stYield: first cutting yield, 2ndYield: second cutting yield, 3rdYield: third cutting yield, 4thYield: fourth cutting yield, Annual Yield: total yield from four cuttings.
